# Tolerability of a single IV administration of a methylene blue challenge in patients with bipolar disorder: preliminary data from a pharmaco-MRI study

**DOI:** 10.1192/bjo.2021.768

**Published:** 2021-06-18

**Authors:** Harriet Sharp, Alfonso Russo, Alessandro Colasanti, Antonello Pinna, Prince Nwaubani, Riccardo De Marco

**Affiliations:** Department of Clinical Neuroscience and Neuroimaging, Brighton and Sussex Medical School, Sussex Partnership NHS Foundation Trust

## Abstract

**Aims:**

To summarise the tolerability profile following an infusion of methylene blue (MB), including subjective effects on mood and energy levels and haemodynamic changes, in patients with Bipolar Affective Disorder (BPAD).

**Background:**

BPAD is associated with mitochondrial dysfunction and impaired cellular energy production. MB is proposed to enhance mitochondria function via rerouting electrons and intracellular reduction of oxidative stress, and is therefore a candidate compound for use as a probe to reveal alterations in brain oxygen metabolism in vivo in patients with BPAD. Although there are reports of MB used as treatment for BPAD, the tolerability and subjective effects of a single IV dose in this population has not yet been defined.

**Method:**

Using a single-blind, randomised, within-subject design, 7 patients with BPAD on stable pharmacological treatment and 6 healthy controls (HCs) received an infusion of 0.5mg/kg MB and a placebo glucose solution one week apart. Visual Analogue Scales (VAS) assessing ‘Mood’ and ‘Energy’ levels were completed by 11 participants, and blood pressure (BP), heart rate (HR) and any subsequent side effects were recorded before and after infusions.

**Result:**

A significant, albeit very small, effect of MB on ‘Mood’ levels relative to placebo was demonstrated, independent of groups (change relative to baseline: 5.5% ± 11 increase (placebo) vs -1.6 % ± 9.5 reduction (MB); p = 0.027). Although there was no effect of MB on energy levels in either group, there appeared to be a trend for a general group difference in ‘Energy’ levels across all trials, with lower ratings in BPAD patients (p = 0.058).

There was a trend for significantly lower post-infusion HR relative to pre-infusion (-6.4 ± 8.8 bpm, p = 0.07. Diastolic BP was higher (3.0 ± 7.8mmHg, p = 0.039). These effects were independent of groups and drug. The most common side effect with MB was mild/moderate pain at infusion site (n = 10/13), resolving within median 32.5 minutes (IQR 6-102), and discoloured urine in 7/13 subjects lasting median 44.5 hours (IQR 36-59). No difference in frequency of side effects reported between groups.

**Conclusion:**

Although limited by small sample size, this tolerability analysis demonstrates a acceptable profile of effects of MB on subjective ratings and blood pressure, in both BPAD and HCs. Common side effects of discoloured urine and pain at infusion site are in line with previous reports in the literature. We observed a small effect of MB on mood ratings which could be related to the discomfort experienced during infusion.

